# Influence of activity space on the association between neighborhood characteristics and dementia risk: results from the 3-City study cohort

**DOI:** 10.1186/s12877-018-1017-7

**Published:** 2019-01-07

**Authors:** Noémie Letellier, Isabelle Carrière, Laure-Anne Gutierrez, Audrey Gabelle, Jean-François Dartigues, Carole Dufouil, Catherine Helmer, Emmanuelle Cadot, Claudine Berr

**Affiliations:** 1 INSERM, University Montpellier, Neuropsychiatry: Epidemiological and Clinical Research, Montpellier, France; 2 Department of Neurology, Memory Research and Resources Center, Montpellier University Hospital Gui de Chauliac, F-34295 Montpellier, France; 3 Inserm, Bordeaux Population Health Research Center, team SEPIA, UMR 1219, University Bordeaux, F-33000 Bordeaux, France; 4 CHU Bordeaux, CMRR, F-33000 Bordeaux, France; 5 Bordeaux school of public health (ISPED), Inserm, Bordeaux Population Health Research Center, team VINTAGE, UMR 1219, University Bordeaux, F-33000 Bordeaux, France; 6 Inserm, Bordeaux Population Health Research Center, team LEHA, UMR 1219, University Bordeaux, F-33000 Bordeaux, France; 7 IRD – Hydrosciences UMR 5569, Montpellier University, F-34090 Montpellier, France

**Keywords:** Cognitive aging, Social health inequalities, Living environment, Deprivation, Life-space mobility

## Abstract

**Background:**

Socioeconomic level of residential environment was found to influence cognitive performance. However, individuals from the same place of residence may be affected differently. We aim to investigate for the first time the influence of individual activity space on the association between neighborhood socioeconomic status (NSES) and the risk of dementia.

**Methods:**

In the frame of the Three-City cohort, a French population-based study, we followed longitudinally (12 years) 7009 participants aged over 65. The activity space (i.e., the spatial area through which a person moves daily) was defined using two questions from Lawton’s Instrumental Activities of Daily Living scale (“Goes shopping independently”,“Travels alone”), and one question about mobility restriction. The survival analysis was performed using a Cox marginal model that takes into account intra-neighborhood correlations and includes a large number of potential confounders.

**Results:**

Among people with a limited activity space (*n* = 772, 11%), risk of dementia is increased in subjects living in a deprived area (characterized by high GINI index or low median income) compared to those living in more favored.

**Conclusion:**

This study shows that the individual activity space modifies the association between NSES and the risk of dementia providing a more complete picture of residential inequalities. If confirmed in different populations, these findings suggest that people with limited activity space and living in a deprived neighborhood are particularly at risk and should be targeted for prevention.

## Background

Social inequalities in health can be determined by place of residence [1, 2]. Recent studies suggested that the living environment might influence cognitive aging [3–8] and the risk of dementia [9, 10]. However, some individual characteristics could modify the impact of neighborhood characteristics on cognition [11], such as ethnicity [12, 13] or social class [14]. For instance, we showed that the risk of dementia is increased only in women living in the most deprived neighborhoods [9]. Such effect of contextual risk factors on dementia incidence only in women might be related by their greater propensity to perform their activities in their neighborhood of residence. Indeed, women of this generation are less likely to work and to have a driving license, and live more often alone.

In order to explore this assumption, we defined the construct of activity space. Activity space has been defined as “the subset of all locations within which an individual has direct contact as a result of his or her day-to-day activities” [15]. It is a measure of daily mobility, and reflects the spatial area through which a person moves over a specific time period [16]. Activity space may better reflect the individual exposure to the living environment because it captures the spatial range of daily experience [17, 18]. People move in and out of their residential neighborhood in the course of their daily activities, and may encounter different types and levels of resources [19]. Whether the activity space could modulate the neighborhood influence on cognition is still unknown.

Based on the hypothesis that the activity space might contribute to inequalities in the contextual exposure to deprivation and resource access [17, 20], we investigated whether activity space modifies the relation between living environment and dementia risk in a cohort of community-living older people.

## Methods

### Study population

Data were analyzed in 2018 from the Three-City Study (3C), a longitudinal community-living cohort of people aged 65 years and over included from the electoral rolls of three French cities (Bordeaux, Dijon, and Montpellier) between 1999 and 2001. The 3C study main objective [21] was to assess the risk of dementia and cognitive impairment related to vascular factors.

Among the 9294 participants, we selected those with identifiable geographical area of residence and data on environmental exposure, and we restricted the analyses to geographical areas where at least five participants were living (*n* = 8457). We also excluded 213 subjects with prevalent dementia, 816 without follow-up, and 419 subjects with missing data for individual covariates. Finally, we included 7009 individuals in the analysis.

Each participant signed an informed consent. The study protocol was approved by the Ethics Committees of the Hospital of Kremlin-Bicêtre and Sud-Méditerranée III.

### Activity space

We created the activity space proxy on an empirical basis and expert advice, using three questions included in the 3C protocol. This proxy indicated the degree of mobility within the living space. The first two questions were from the Lawton’s Instrumental Activities of Daily Living (IADL) scale (“Goes shopping independently” and “Travels alone using different means of transport”) [22]. The last one (“Do you have trouble moving?”) was from the mobility scale and had four possible answers (“Confined to bed or chair”; “Confined at home”; “Confined to the neighborhood”, and “No restriction”).

We considered individuals as having a limited activity space when they needed help to go shopping, or were unable to move without being accompanied, or were confined at home or to their neighborhood; otherwise, we classified them as having an unlimited activity space.

### Neighborhood socio-economic status (NSES)

Based on geocoding of postal addresses of participants, we matched them to their IRIS neighborhood of residence [9, 23]. IRIS is the smallest and most detailed census aggregation level employed by the French National Institute of Statistics and Economic Studies to disseminate information (i.e., “Ilots Regroupés pour l’Information Statistique”, IRIS).

We used data from the 1999 census and the 2001 “household tax income” to evaluate the NSES at baseline. The NSES is generally regarded as the combination of socioeconomic variables at the individual or household level, and is often assessed using a poverty index. We previously defined a 3C deprivation score [9] by Principal Component Analysis. The 3C deprivation score was characterized by a positive score and high weight for the following components: proportion of households without a car, of tenants and single parents, Gini index (an indicator of income inequality), unemployment rate, and settlement index; and a negative score for the tax household income. The IRIS neighborhood position on this axis defines its degree of deprivation. We categorized the 3C deprivation score in tertiles (T1, T2 and T3; from the least to the most deprived neighborhood) [9].

### Diagnosis of dementia

First, at baseline and each follow-up, the evaluation of neuropsychological tests (Mini-Mental State 130 Examination (MMSE), the Isaacs Set Test, and the Benton Visual Retention Test) is performed by trained psychologist. Participants underwent dementia screening with neuropsychological exams during the 12-year follow-up of the 3C study. As detailed previously [9], diagnosis of dementia was assessed through two- (all subjects examined by a neurologist) or three- (selection of subject according to neuropsychological battery results) step procedure according to the center and time of exam. The final step was the case review by an independent committee of neurologists to obtain a consensus on the diagnosis according to the DSM-IV criteria [24]. For the analyses, we considered all incident cases of all-cause dementia over the 12-year follow-up period.

### Other variables

We evaluated the individual socio-economic status (SES) using the following variables: sex, age, study center, level of education (primary, secondary and higher), monthly household income (≥2287€ and < 2287€) and former occupational category (blue collars: workers, farmers, artisans; and white collars). We also considered behavior variables and vascular risk factors as potential confounders: alcohol consumption (non-consumer; 1–36 g/day; > 36 g/day), smoking status (current smoker; former smoker; non-smoker), body mass index (BMI) categories (underweight: BMI < 18.5 kg/m^2^; normal: 18.5 ≤ BMI < 25 kg/m^2^; overweight: 25 ≤ BMI < 30 kg/m^2^; obesity: BMI ≥ 30 kg/m^2^), diabetes (antidiabetic treatment, or glycemia > 7.0 mmol/L, or diabetes history), hypertension (systolic blood pressure > 140 mmHg or diastolic blood pressure > 90 mmHg, or antihypertensive drug intake), hypercholesterolemia (fasting total cholesterol > 6.2 mmol/L, or lipid-lowering drug intake), and APOEε4 carrier (defined as the presence of at least one ε4 allele). We also included self-reported history of cardiovascular diseases (CVD) (including stroke, angina pectoris, myocardial infarction and cardiac and vascular surgery), depressive symptoms (Center for Epidemiologic Studies Depression Scale score ≥ 17 for men and ≥ 23 for women, or too depressed to respond), and IADL limitations defined using the three questions of the Lawton scale shared by both sexes and not included in the activity space proxy: ability to use the phone, responsibility for taking medications and ability to manage the budget (IADL «budget, medication, phone»).

To investigate social isolation, we recorded whether the person lived alone (yes/no), and used a social network index (SNI) for the sensitivity analyses. The 3C SNI was inspired by the Berkman-Syme Social Network Index [25], and was available only for the Montpellier and Dijon centers (5083 participants). This is a composite measure of three types of social connections: marital status (no: 0; yes: 1), sociability/contacts with close friends and relatives (never or sometimes: 0; regularly or often: 1), and participation in organization(s)/club(s) (never or almost never: 0; all other options: 1). Based on the total SNI score, we defined a person as socially isolated (score = 0), moderately isolated (score = 1), moderately integrated (score = 2), and socially integrated (score = 3).

### Statistical analysis

We performed longitudinal analysis to study all variables associated with the risk of dementia using a marginal Cox model with age as the time scale [26]. This model, which uses a robust sandwich variance estimator, takes into account the correlations between individuals in the same geographical area. We conducted univariate and multivariate analyses. We selected individual covariates for multivariate analysis by combining information from univariate analyses (selection of variables with *p* < 0.20) and literature [27, 28]. Therefore, for the multivariate analyses, we retained individual sociodemographic variables (sex, study center, educational level, income and occupational grade) and health status variables (APOEε4, diabetes, cardiovascular disease, depressive symptoms and IADL «budget, medication, phone»). We tested the interactions between activity space and NSES determinants or sex. In view of our previous results [9], we decided to perform additional analyses restricted to women.

We expressed results as hazard ratios (HR) and 95% confidence intervals (CI). We used the Cochran-Armitage trend test to analyze the dose-effect, when appropriate. We used the SAS (SAS version 9.4) procedure PHREG to estimate the parameters. In a subsample analyses, we used chi-square test to evaluate the association between activity space and 3C SNI.

## Results

### Subjects characteristics

Among the 7009 people retained for this study (54,857 person-years (py)), 789 developed incident dementia over the 12-year follow-up period, corresponding to an annual incidence rate of 14.4/1000 py. The median age at enrollment was 73.5 years and participants had been living in the same residence for 25 years on average (SD 15). Among the 7009 participants, 62% were women, 37% lived alone, 24% had only primary education, 18% were blue collars, 13% had depressive symptoms, 38% were current smokers or former smokers, 52% were overweight or obese, 9% had diabetes and 9% had history of CVD. Among the 789 people who developed dementia, 554 had Alzheimer’s dementia (68.7%).

### Individual characteristics according to the activity space

Activity space was limited in 11% of participants (*n* = 772). Compared with participants with unlimited activity space, people with limited activity space were older, more often women and widowed, and frequently lived alone (Table 1). They were less likely to consume alcohol and tobacco. Conversely, underweight, diabetes, CVD history, hypertension and depression were more frequent in people with limited activity space, as well as dependency for daily activities (8.0% vs 0.8% for unlimited activity space). Dementia incidence also was higher among people with limited activity space (38.4/1000py vs 12.2/1000 py for unlimited activity space).

**Table 1:** Distribution of individual baseline characteristics according to activity space

Individual characteristics, N (%)	No limited activity space (*n* = 6237)	Limited activity space (*n* = 772)	p^a^
Socio-demographic and socio-economic factors			
Female	3735 (59.9)	591 (76.6)	<.0001
Age at inclusion (years)^b^	73.6 (5.1)	78.4 (6.1)	<.0001
Study center			<.0001
Bordeaux	1362 (21.8)	223 (28.9)	
Dijon	3641 (58.4)	439 (56.9)	
Montpellier	1234 (19.8)	110 (14.3)	
Familial status (*n* = 6988)			<.0001
Divorced or single	906 (14.6)	126 (16.3)	
Married	3737 (60.2)	346 (44.9)	
Widowed	1567 (25.2)	299 (38.8)	
Primary study	1407 (22.6)	266 (34.5)	<.0001
Income ≥2287 €	2169 (34.8)	145 (18.8)	<.0001
Blue collars	1072 (17.2)	189 (24.5)	<.0001
Living alone (*n* = 6992)	2217 (35.6)	372 (48.4)	<.0001
IADL « budget, medication, phone »	51 (0.8)	62 (8.0)	<.0001
Factors related to lifestyle			
Alcohol consumption (*n* = 6901)			
Non consumer	1148 (18.7)	254 (33.5)	<.0001
1-36 g/day	4474 (72.9)	474 (62.5)	
> 36 g/day	513 (8.4)	31 (4.1)	
Smoking status (*n* = 7013)			
Current smoker	342 (5.5)	29 (3.8)	<.0001
Former smoker	2112 (33.9)	186 (24.2)	
Non-smoking	3782 (60.7)	555 (72.1)	
Factors related to health			
Presence of the APOEε4	1250 (20.0)	140 (18.1)	0.21
Body mass index			<.0001
Underweight (< 18.5)	123 (2.0)	58 (7.5)	
Normal (18.5–25)	2869 (46.0)	290 (37.6)	
Overweight [25–30]	2459 (39.4)	274 (35.5)	
Obese (> 30)	786 (12.6)	150 (19.4)	
Diabetes	536 (8.6)	112 (14.5)	<.0001
History of CVD	508 (8.1)	118 (15.3)	<.0001
Hypertension^c^ (*n* = 6858)	2499 (40.9)	386 (51.8)	<.0001
Hypercholesterolemia^d^ (*n* = 7000)	3580 (57.5)	406 (52.7)	0.0123
Depressive syndrome (*n* = 6924)	697 (11.2)	193 (25.0)	<.0001
Incidence rate of dementia (/1000 py)	12.2	38.4	

^a^Wilcoxon test for age, chi-square test for other variables

^b^MEAN (SD)

^c^Hypertension: systolic blood pressure > 140 mmHg or diastolic blood pressure > 90 mmHg or antihypertensive drug intake)

^d^Hypercholesterolemia: fasting total cholesterol> 6.2 mmol/L or lipid-lowering drug intake

CVD, cardiovascular diseases; IADL, Instrumental Activities of Daily Living

Note: People with limited activity space = people who have need to help to go shopping, or if they are unable to move without being accompanied, or if people are confined at home or at their neighborhood

### Activity space and dementia

In univariate models, individuals with limited activity space (to move, shopping or use public transport) were at greater risk of developing dementia (HR = 2.06, 95% CI = 1.75–2.42), compared with individuals with unlimited activity space. After adjusting for socioeconomic (sex, study center, educational level, income and occupational grade) and health characteristics (APOEε4, diabetes, cardiovascular disease, depressive symptoms and IADL «budget, medication, phone»), this risk decreased but remained significant (adjusted HR = 1.55, 95% CI = 1.31–1.84).

#### Deprived or unequal neighborhoods, activity space and dementia

Interactions between some neighborhood composition indicators and activity space were detected: 3C deprivation score (*p* = 0.07), proportion of blue-collar workers (*p* = 0.14), of households without car (*p* = 0.03), and of people aged 60 years or over (*p* = 0.06), Gini index (*p* = 0.04) and median household net taxable income (*p* = 0.03). These analyses did not highlight any interaction between activity space and sex.

These neighborhood composition indicators modulated the risk of dementia mainly when activity space was limited. Specifically, the 3C deprivation score was associated with the risk of incident dementia only in people with limited activity space. The number of incident dementia cases was lower (13.2/1000py) in the least deprived neighborhoods (T1), and progressively increased with the neighborhood deprivation (13.9/1000py in the intermediate (T2), and 16.0/1000py in the most deprived neighborhoods (T3)). In univariate analyses (with age as baseline time), the risk of dementia was significantly increased only for the most disadvantaged neighborhoods compared with the least disadvantaged (T3 HR = 1.45, 95% CI 1.01–2.06), but was no longer significant after adjustments for confounders (Table 2). Moreover, the dementia risk was higher for people who lived in neighborhoods with high proportion of car-free households (> 29.0%; T3) than for those in neighborhoods with low proportion (< 21.2%; T1), but only if their activity space was limited. This association remained significant after adjusting for individual SES and health status variables (T3 adjusted HR = 1.42, 95% CI 1.00–2.03) (Table 2). People with limited activity space and residing in neighborhoods with high Gini index, where income inequalities were higher, also had a higher risk of dementia (T3 adjusted HR = 1.60, 95% CI 1.04–2.45) (Table 2). Compared with those living in a neighborhood where the proportion of blue-collar workers was lower than 13.6% (T1), living in a neighborhood with a high proportion of blue-collar workers (> 20.8%; T3) also was associated with a higher risk of dementia only for individuals with limited activity space (T3 HR = 1.38, 95% CI = 1.00–1.90) After adjustment for individual characteristics, this association was no longer significant (T3 adjusted HR = 1.19, 95% CI 0.85–1.67) (Table 2).

**Table 2:** Association between deprived and unequal neighborhood characteristics and risk of dementia, according to activity space

*Deprived and unequal neighborhood characteristics*		All-type dementia (*n* = 789)	Univariate model		Multivariate model^a^	
		n	HR (95% IC)	p	HR (95% IC)	p
3C deprivation score	*No limited activity space*					
	T1 (most privileged)	192	1	–	1	–
	T2	202	0.97 (0.79–1.18)	0.73	0.97 (0.80–1.17)	0.74
	T3 (most deprived)	218	1.06 (0.85–1.31)	0.62	1.04 (0.83–1.29)	0.76
	Global *p*-value			0.64		0.80
	*Limited activity space*					
	T1 (most privileged)	47	1	–	1	–
	T2	51	1.09 (0.73–1.63)	0.68	1.07 (0.72–1.60)	0.74
	T3 (most deprived)	79	**1.45 (1.01–2.06)**	**0.04**	1.37 (0.92–2.05)	0.12
	Global p-value			0.06		0.23
Proportion of blue collar workers	*No limited activity space*					
	T1 (< 13.6)	215	1	–	1	–
	T2 (13.6–20.8)	186	0.98 (0.81–1.18)	0.81	0.90 (0.75–1.08)	0.24
	T3 (> 20.8)	211	1.13 (0.93–1.38)	0.21	0.95 (0.78–1.15)	0.61
	*Global p-value*			0.32		0.51
	*Limited activity space*					
	T1 (< 13.6)	50	1	–	1	–
	T2 (13.6–20.8)	52	1.01 (0.71–1.44)	0.96	0.94 (0.65–1.35)	0.73
	T3 (> 20.8)	75	**1.38 (1.00–1.90)**	**0.05**	1.19 (0.85–1.67)	0.32
	*Global p-value*			0.09		0.37
Proportion of households without car	*No limited activity space*					
	T1 (< 21.2)	188	1	–	1	–
	T2 (21.2–29.0)	221	1.17 (0.96–1.43)	0.13	**1.20 (1.00–1.44)**	**0.05**
	T3 (> 29.0)	203	1.00 (0.81–1.24)	0.99	1.00 (0.83–1.22)	0.98
	*Global p-value*			0.19		0.08
	*Limited activity space*					
	T1 (< 21.2)	44	1	–	1	–
	T2 (21.2–29.0)	56	1.28 (0.86–1.90)	0.22	1.36 (0.92–2.01)	0.13
	T3 (> 29.0)	77	**1.48 (1.04–2.12)**	**0.03**	**1.42 (1.00–2.03)**	**0.05**
	*Global p-value*			0.09		0.14
Gini index	*No limited activity space*					
	T1 (< 0.31)	191	1	–	1	–
	T2 (0.31–0.35)	214	1.05 (0.85–1.29)	0.64	1.09 (0.89–1.33)	0.42
	T3 (> 0.35)	207	0.93 (0.75–1.15)	0.48	0.97 (0.78–1.20)	0.75
	*Global p-value*			0.39		0.42
	*Limited activity space*					
	T1 (< 0.31)	48	1	–	1	–
	T2 (0.31–0.35)	62	1.23 (0.82–1.84)	0.32	1.28 (0.86–1.89)	0.22
	T3 (> 0.35)	67	1.45 (0.98–2.15)	0.06	**1.60 (1.04–2.45)**	**0.03**
	Global *p*-value			0.16		0.10

^a^Marginal Cox model adjusted for sex, study center, education level, income, occupational category, APOEε4 carrier status, diabetes, history of cardiovascular diseases, depressive symptoms and disability (IADL « budget, medication, phone »)

Note: People with limited activity space = people who have need to help to go shopping, or if they are unable to move without being accompanied, or if people are confined at home or at their neighborhood

#### Advantaged neighborhoods, activity space and dementia

The risk of dementia was decreased for people with a limited activity space only when they lived in quite wealthy neighborhoods, where the median household net taxable income per consumption unit was higher than 15,500 € (T2 HR = 0.68, 95% CI 0.49–0.94; T3 HR = 0.64, 95% CI 0.46–0.90), even after adjustment (T2 adjusted HR = 0.67, 95% CI = 0.48–0.94; T3 adjusted HR = 0.68, 95% CI = 0.46–1.00) (Table 3). The risk of dementia for individuals with limited activity was reduced also when they lived in a neighborhood with higher proportion of people over 60 years (T2 adjusted HR = 0.65, 95% CI = 0.47–0.91; T3 adjusted HR = 0.71, 95% CI = 0.51–0.99) (Table 3).

**Table 3:** Association between advantaged neighborhood characteristics and risk of dementia, according to activity space

*Advantaged neighborhood characteristics*		All-type dementia (*n* = 789)	Univariate model		Multivariate model^a^	
		n	HR (95% IC)	p	HR (95% IC)	p
Median household net taxable income	*No limited activity space*					
	T1 (< 15,487)	210	1	–	1	–
	T2 (15487–18,091)	202	1.00 (0.81–1.24)	0.99	1.06 (0.86–1.31)	0.58
	T3 (> 18,091)	200	0.92 (0.76–1.10)	0.34	1.01 (0.83–1.24)	0.91
	Global p-value			0.55		0.83
	*Limited activity space*					
	T1 (< 15,487)	81	**1**	**–**	**1**	**–**
	T2 (15487–18,091)	53	**0.68 (0.49–0.94)**	**0.02**	**0.67 (0.48–0.94)**	**0.02**
	T3 (> 18,091)	43	**0.64 (0.46–0.90)**	**0.009**	**0.68 (0.46–1.00)**	**0.05**
	Global p-value			**0.008**		**0.03**
Proportion of people aged 60 years or over	*No limited activity space*					
	T1 (< 20.0)	214	1	–	1	–
	T2 (20.0–24.7)	190	0.89 (0.73–1.09)	0.26	0.93 (0.77–1.14)	0.49
	T3 (> 24.7)	208	0.97 (0.79–1.19)	0.78	0.97 (0.80–1.19)	0.78
	Global p-value			0.48		0.78
	*Limited activity space*					
	T1 (< 20.0)	79	**1**	**–**	**1**	**–**
	T2 (20.0–24.7)	42	**0.61 (0.44–0.85)**	**0.003**	**0.65 (0.47–0.91)**	**0.01**
	T3 (> 24.7)	56	**0.68 (0.49–0.95)**	**0.02**	**0.71 (0.51–0.99)**	**0.04**
	Global *p*-value			**0.004**		**0.02**

^a^Marginal Cox model adjusted for sex, study center, education level, income, occupational category, APOEε4 carrier status, diabetes, history of cardiovascular diseases, depressive symptoms and disability (IADL « budget, medication, phone »)

Note: People with limited activity space = people who have need to help to go shopping, or if they are unable to move without being accompanied, or if people are confined at home or at their neighborhood

### Complementary analyses

When we restricted these analyses to women only, we did not find the negative effect of disadvantaged neighborhoods for the limited activity space group (T3 3C deprivation score HR = 1.49, 95% CI = 0.96–2.30) (Table 5). Conversely, we confirmed the protective effect of advantaged neighborhood characteristics (median income and proportion of people aged 60 years or over) in the case of limited activity space (T3 HR = 0.61, 95% CI = 0.41–0.89; and T3 adjusted HR = 0.68, 95% CI = 0.48–0.96, respectively) (Table 6).

Other complementary analyses on a subsample with available data (participants from Montpellier and Dijon) showed that the activity space was associated with the 3C SNI (Table 4). Specifically, people with limited activity space were more socially isolated (10.8% vs 5.2%, *p* < 0.0001). The influence of the activity space on social isolation remained significant after adjustment for physical activity.

**Table 4:** Distribution of social isolation (SNI 3C) according to activity space in a subsample (*n* = 5083)

N (%)	No limited activity space (*n* = 4592)	Limited activity space (*n* = 491)	p*
Social Network index 3C			<.0001
Socially isolated	237 (5.2)	53 (10.8)	
Moderatly isolated	1268 (27.6)	198 (40.3)	
Moderatly integrated	2063 (44.9)	183 (37.3)	
Socially integrated	1024 (22.3)	57 (11.6)	

*Chi-square test

**Table 5:** Association between deprived and unequal neighborhood characteristics and risk of dementia only in women

*Deprived and unequal neighborhood characteristics*		All-type dementia (*n* = 517)	Univariate model		Multivariate model^a^	
		n	HR (95% IC)	p	HR (95% IC)	p
3C deprivation score	*No limited activity space*					
	T1 (most privileged)	110	1	–	1	–
	T2	120	1.03 (0.78–1.35)	0.85	1.01 (0.79–1.30)	0.91
	T3 (most deprived)	151	1.24 (0.97–1.60)	0.09	1.25 (0.98–1.60)	0.08
	*Limited activity space*					
	T1 (most privileged)	36	1	–	1	–
	T2	40	1.07 (0.68–1.73)	0.68	1.08 (0.68–1.70)	0.76
	T3 (most deprived)	60	1.49 (0.96–2.30)	0.08	1.39 (0.89–2.16)	0.15
Proportion of blue collar workers	*No limited activity space*					
	T1 (< 13.6)	135	1	–	1	–
	T2 (13.6–20.8)	119	0.98 (0.74–1.29)	0.87	0.87 (0.68–1.10)	0.24
	T3 (> 20.8)	127	1.06 (0.83–1.36)	0.63	0.87 (0.69–1.09)	0.23
	*Limited activity space*					
	T1 (< 13.6)	42	1	–	1	–
	T2 (13.6–20.8)	39	0.92 (0.62–1.38)	0.68	0.93 (0.61–1.40)	0.72
	T3 (> 20.8)	55	1.21 (0.84–1.74)	0.32	1.11 (0.78–1.60)	0.56
Proportion of households without car	*No limited activity space*					
	T1 (< 21.2)	110	1	–	1	–
	T2 (21.2–29.0)	137	1.14 (0.89–1.46)	0.30	1.19 (0.96–1.49)	0.12
	T3 (> 29.0)	134	1.10 (0.84–1.46)	0.49	1.11 (0.86–1.44)	0.43
	*Limited activity space*					
	T1 (< 21.2)	34	1	–	1	–
	T2 (21.2–29.0)	41	1.20 (0.75–1.91)	0.46	1.29 (0.82–2.02)	0.27
	T3 (> 29.0)	61	1.43 (0.91–2.25)	0.12	1.46 (0.94–2.26)	0.09
Gini index	*No limited activity space*					
	T1 (< 0.31)	113	1	–	1	–
	T2 (0.31–0.35)	133	1.09 (0.84–1.41)	0.53	1.10 (0.87–1.41)	0.43
	T3 (> 0.35)	135	1.04 (0.80–1.35)	0.80	1.14 (0.89–1.45)	0.30
	*Limited activity space*					
	T1 (< 0.31)	39	1	–	1	–
	T2 (0.31–0.35)	47	1.25 (0.78–2.00)	0.35	1.32 (0.86–2.04)	0.22
	T3 (> 0.35)	50	1.36 (0.86–2.16)	0.19	1.28 (0.82–2.01)	0.28

^a^Marginal Cox model adjusted for study center, education level, income, occupational category, APOEε4 carrier status, diabetes, history of cardiovascular diseases, depressive symptoms and disability (IADL « budget, medication, phone »)

Note: People with limited activity space = people who have need to help to go shopping, or if they are unable to move without being accompanied, or if people are confined at home or at their neighborhood

**Table 6:** Association between advantaged neighborhood characteristics and risk of dementia only in women

*Advantaged neighborhood characteristics*		All-type dementia (*n* = 517)	Univariate model		Multivariate model^a^	
		n	HR (95% IC)	p	HR (95% IC)	p
Median household net taxable income	*No limited activity space*					
	T1 (< 15,487)	139	1	–	1	–
	T2 (15487–18,091)	125	0.97 (0.75–1.26)	0.83	0.96 (0.76–1.20)	0.70
	T3 (> 18,091)	117	0.85 (0.67–1.09)	0.21	0.89 (0.70–1.14)	0.37
	*Limited activity space*					
	T1 (< 15,487)	59	**1**	**–**	1	–
	T2 (15487–18,091)	45	**0.73 (0.50–1.05)**	**0.09**	0.76 (0.53–1.07)	0.11
	T3 (> 18,091)	32	**0.61 (0.41–0.89)**	**0.01**	**0.67 (0.44–1.02)**	**0.06**
Proportion of people aged 60 years or over	*No limited activity space*					
	T1 (< 20.0)	145	1	–	1	–
	T2 (20.0–24.7)	113	0.80 (0.61–1.04)	0.10	0.85 (0.67–1.09)	0.21
	T3 (> 24.7)	123	0.84 (0.66–1.07)	0.17	0.87 (0.69–1.10)	0.24
	*Limited activity space*					
	T1 (< 20.0)	63	**1**	**–**	**1**	**–**
	T2 (20.0–24.7)	30	**0.59 (0.38–0.89)**	**0.01**	**0.60 (0.39–0.92)**	**0.02**
	T3 (> 24.7)	43	**0.69 (0.47–0.99)**	**0.04**	**0.68 (0.48–0.96)**	**0.03**

^a^Marginal Cox model adjusted for study center, education level, income, occupational category, APOEε4 carrier status, diabetes, history of cardiovascular diseases, depressive symptoms and disability (IADL « budget, medication, phone »)

Note: People with limited activity space = people who have need to help to go shopping, or if they are unable to move without being accompanied, or if people are confined at home or at their neighborhood

## Discussion

The results of our study suggests that the individual activity space, a concept that reflects the local areas within which people move in the course of their daily activities [29], influences vulnerability to the neighborhood environment. Specifically, in our cohort of people older than 65 years of age, the risk of dementia was higher among those living in deprived neighborhood only if their activity space was limited. On the other hand, an advantaged neighborhood was associated with a lower risk of dementia only for people with limited activity space, and individual characteristics only slightly changed this effect.

People with a limited activity space are more exposed to risk factors of cognitive decline, such as depressive symptoms [30] or poorer physical activity [31]. In our study, the health status and individual socioeconomic variables reduced the strength of the association between contextual features and dementia risk. However, this association remained significant for several NSES indicators, suggesting that such risk is not fully explained by socioeconomic individual factors and medical problems.

To our knowledge, three previous studies found that greater activity space is associated with reduced cognitive decline, but none examined its influence on the risk of dementia [32–34]. It is not fully understood how activity space can influence cognition [35]. Activity space modulates the relationship with the environment, the daily access to resources, and participation in social, cultural, recreational and physical activities [36, 37]. Furthermore, unrestricted activity space correlates positively with quality of life [38] and active social participation [39, 40]. Conversely, a restricted life-space mobility may decrease social integration, whereas the maintenance of social participation contributes to successful aging [41–43].

Moreover, a deprived residential environment is associated with poorer mental health [44]. It can also exert a stronger influence on the cognitive decline of people whose activity space is limited to their neighborhood of residence, possibly because they are more present and consequently the neighborhood’s influence is greater. Conversely, an advantaged neighborhood of residence is beneficial for health and facilitates cognitive stimulation. Hand and Howrey showed that a higher proportion of neighborhood residents aged 65 and older is associated with increased odds of more frequent participation in social activities, such as club attendance [45], and this can reduce the risk of dementia [46].

To our knowledge, only another study showed that the activity space influences the association between neighborhood of residence and mental health. Vallée et al. found that people living in more deprived neighborhoods are significantly more depressed that those living in more advantaged neighborhood [20]. Other studies showed that taking into account the activity space increases the magnitude of the association between living environment and self-rated health or health screening. A higher exposure to less disadvantaged non-residential neighborhoods during the daily activities is associated with a proportionally better self-rated health [47]. Women living in low medical-density neighborhoods have a significantly higher risk of delayed health screening, but only those who concentrate their daily activities within their neighborhood of residence [48].

The design of this study cohort is one of the main strength of the present study. The 3-City cohort is a large population-based sample with long follow-up and active search of dementia cases, validated by adjudication committee. Furthermore, this study gathered major individual SES variables and documented health status and health risk factors, which gave more strength and confidence in our multivariate analyses results. The results are in line with those previously reported for women [9], although interactions between activity space and sex were not significant, certainly due to the lack of statistical power in the sub-groups. Despite this small subsample, in women only, we confirmed the protective effect of advantaged neighborhood characteristics in the case of limited activity space but do not evidenced the deleterious effect of deprivation.

Our study also has some limitations. First, our population sample is recruited in three French urban areas limiting the generalization of our results. Comparison between subjects included in analyses and those excluded for absence of follow-up or missing data, show that the later lived more frequently in disadvantaged neighborhoods and had poorer health status. The multiplicity of tests due to different neighborhood variables may increase the risk of Type 1 error but each contextual variables represent a different dimension even if some are correlated. Furthermore, the concept of activity space is often assessed using geographic information system methods, and sometimes with the Life-Space Assessment (LSA) questionnaire [49], to score the distance and frequency of movement and assistance needed in moving. However, we did not have access to this kind of data. Therefore, we chose to create a simple and original measure of activity space, by combining two questions of the Lawton’s IADL scale and one about mobility restriction. Activity space represented a major construct in this study. The documentation of activity space is based on existing data sources (IADL-scores) which have not been implemented in the basic study for this purpose and therefore represent a surrogate marker. Our results remained significant when adjusted for disability (the other part of the IADL scale), indicating that our activity space variable goes beyond the loss of autonomy; it is more a “loss of neighborhood”. Overall, as detailed in this discussion, some of our results are difficult to interpret and require further quantitative and qualitative studies.

## Conclusion

For individuals with limited activity space, living in a deprived neighborhood is detrimental for cognitive ageing, whereas living in an advantaged neighborhood is associated with a lower risk of dementia. Activity space may provide a more complete picture of the inequalities induced by residential neighborhood exposure [50]. If confirmed in different populations, these findings suggest that people with limited activity space and living in a deprived neighborhood are particularly at risk and should be targeted for prevention.

## Abbreviations

3C study: Three-City Study
CI: Confidence Intervals
CVD: Cardiovascular Diseases
HR: Hazard Ratios
IADL scale: Instrumental Activities of Daily Living (IADL) scale
IRIS: Ilots Regroupés pour l’Information Statistique (French Abbreviation)
NSES: Neighborhood Socioeconomic Status
SES: Socio-Economic Status
SNI: Social Network Index

## References

[1] Pickett K, Pearl M (2001). Multilevel analyses of neighbourhood socioeconomic context and health outcomes: a critical review. J Epidemiol Community Health.

[2] Tost H, Champagne FA, Meyer-Lindenberg A (2015). Environmental influence in the brain, human welfare and mental health. Nat Neurosci.

[3] Wight RG, Aneshensel CS, Miller-Martinez D, Botticello AL, Cummings JR, Karlamangla AS (2006). Urban neighborhood context, educational attainment, and cognitive function among older adults. Am J Epidemiol.

[4] Sheffield KM, Peek MK (2009). Neighborhood context and cognitive decline in older Mexican Americans: results from the Hispanic established populations for epidemiologic studies of the elderly. Am J Epidemiol.

[5] Wee LE, Yeo WX, Yang GR, Hannan N, Lim K, Chua C (2012). Individual and area level socioeconomic status and its association with cognitive function and cognitive impairment (low MMSE) among community-dwelling elderly in Singapore. Dement Geriatr Cogn Disord Extra.

[6] Sisco SM, Marsiske M (2012). Neighborhood influences on late life cognition in the ACTIVE study. J Aging Res.

[7] Basta NE, Matthews FE, Chatfield MD, Brayne C, MRC-CFAS (2008). Community-level socio-economic status and cognitive and functional impairment in the older population. Eur J Pub Health.

[8] Wu Y-T, Prina AM, Brayne C (2015). The association between community environment and cognitive function: a systematic review. Soc Psychiatry Psychiatr Epidemiol.

[9] Letellier N, Gutierrez L-A, Carrière I, Gabelle A, Dartigues J-F, Dufouil C (2018). Sex-specific association between neighborhood characteristics and dementia: the Three-City cohort. Alzheimers Dement.

[10] Cadar D, Lassale C, Davies H, Llewellyn DJ, Batty GD, Steptoe A. Individual and Area-Based Socioeconomic Factors Associated With Dementia Incidence in England: Evidence from a 12-year follow-up in the English longitudinal study of ageing. JAMA psychiatry. Published online 2018. 10.1001/jamapsychiatry.2018.1012 PMC6145673 29799983

[11] Cassarino M, Setti A (2015). Environment as « Brain Training »: A review of geographical and physical environmental influences on cognitive ageing. Ageing Res Rev.

[12] Espino DV, Lichtenstein MJ, Palmer RF, Hazuda HP (2001). Ethnic differences in mini-mental state examination (MMSE) scores: where you live makes a difference. J Am Geriatr Soc.

[13] Aneshensel CS, Ko MJ, Chodosh J, Wight RG (2011). The urban neighborhood and cognitive functioning in late middle age. J Health Soc Behav.

[14] Deeg DJH, Thomése GCF (2005). Discrepancies between personal income and neighbourhood status: effects on physical and mental health. Eur J Ageing.

[15] Golledge RG, Stimson RJ (1997). Spatial Behavior: A geographic perspective.

[16] Sherman JE, Spencer J, Preisser JS, Gesler WM, Arcury TA (2005). A suite of methods for representing activity space in a healthcare accessibility study. Int J Health Geogr.

[17] Jones M, Pebley AR (2014). Redefining neighborhoods using common destinations: social characteristics of activity spaces and home census tracts compared. Demography.

[18] Perchoux C, Chaix B, Cummins S, Kestens Y (2013). Conceptualization and measurement of environmental exposure in epidemiology: accounting for activity space related to daily mobility. Health Place.

[19] Basta LA, Richmond TS, Wiebe DJ (2010). Neighborhoods, daily activities, and measuring health risks experienced in urban environments. Soc Sci Med 1982.

[20] Vallée J, Cadot E, Roustit C, Parizot I, Chauvin P (2011). The role of daily mobility in mental health inequalities: the interactive influence of activity space and neighbourhood of residence on depression. Soc Sci Med.

[21] 3C Study Group (2003). Vascular factors and risk of dementia: design of the Three-City study and baseline characteristics of the study population. Neuroepidemiology.

[22] Lawton MP, Brody EM (1969). Assessment of older people: self-maintaining and instrumental activities of daily living. The Gerontologist.

[23] Grimaud O, Lapostolle A, Berr C, Helmer C, Dufouil C, Kihal W (2013). Gender differences in the association between socioeconomic status and subclinical atherosclerosis. PLoS One.

[24] American Psychiatric Association (1994). Diagnostic and statistical manual of mental disorders: DSM-IV.

[25] Berkman LF, Syme SL (1979). Social networks, host resistance, and mortality: a nine-year follow-up study of Alameda County residents. Am J Epidemiol.

[26] Thiébaut ACM, Bénichou J (2004). Choice of time-scale in Cox’s model analysis of epidemiologic cohort data: a simulation study. Stat Med.

[27] Plassman BL, Williams JW, Burke JR, Holsinger T, Benjamin S (2010). Systematic review: factors associated with risk for and possible prevention of cognitive decline in later life. Ann Intern Med.

[28] Kivipelto M, Mangialasche F, Ngandu T (2018). Lifestyle interventions to prevent cognitive impairment, dementia and Alzheimer disease. Nat Rev Neurol.

[29] Gesler WM, Albert DP. How spatial analysis can be used in medical geography. Spat anal GIS remote Sens Appl. Health Sci. 2000:11–38.

[30] Polku H, Mikkola TM, Portegijs E, Rantakokko M, Kokko K, Kauppinen M (2015). Life-space mobility and dimensions of depressive symptoms among community-dwelling older adults. Aging Ment Health.

[31] Eronen J, von Bonsdorff M, Rantakokko M, Portegijs E, Viljanen A, Rantanen T (2016). Socioeconomic status and life-space mobility in old age. J Aging Phys Act.

[32] Silberschmidt S, Kumar A, Raji MM, Markides K, Ottenbacher KJ, Al Snih S (2017). Life-space mobility and cognitive decline among Mexican Americans aged 75 years and older. J Am Geriatr Soc.

[33] Crowe M, Andel R, Wadley VG, Okonkwo OC, Sawyer P, Allman RM (2008). Life-space and cognitive decline in a community-based sample of African American and Caucasian older adults. J Gerontol A Biol Sci Med Sci.

[34] James BD, Boyle PA, Buchman AS, Barnes LL, Bennett DA (2011). Life space and risk of Alzheimer disease, mild cognitive impairment, and cognitive decline in old age. Am J Geriatr Psychiatry Off J Am Assoc Geriatr Psychiatry.

[35] Béland F, Julien D, Bier N, Desrosiers J, Kergoat M-J, Demers L (2018). Association between cognitive function and life-space mobility in older adults: results from the FRéLE longitudinal study. BMC Geriatr.

[36] Hanson S (2005). Perspectives on the geographic stability and mobility of people in cities. Proc Natl Acad Sci U S A.

[37] Rantanen T (2013). Promoting mobility in older people. J Prev Med Pub Health.

[38] Rantakokko M, Portegijs E, Viljanen A, Iwarsson S, Rantanen T (2013). Life-space mobility and quality of life in community-dwelling older people. J Am Geriatr Soc.

[39] Tomioka K, Kurumatani N, Hosoi H (2016). Association between social participation and instrumental activities of daily living among community-dwelling older adults. J Epidemiol.

[40] Barnes LL, Wilson RS, Bienias JL, de Leon CFM, Kim H-JN, Buchman AS (2007). Correlates of life space in a volunteer cohort of older adults. Exp Aging Res.

[41] Rowe JW, Kahn RL (2015). Successful aging 2.0: conceptual expansions for the 21st century. J Gerontol Ser B.

[42] Chiao C, Weng L-J, Botticello AL (2011). Social participation reduces depressive symptoms among older adults: an 18-year longitudinal analysis in Taiwan. BMC Public Health.

[43] James BD, Wilson RS, Barnes LL, Bennett DA (2011). Late-life social activity and cognitive decline in old age. J Int Neuropsychol Soc JINS.

[44] Stafford M, Marmot M (2003). Neighbourhood deprivation and health: does it affect us all equally?. Int J Epidemiol.

[45] Hand CL, Howrey BT. Associations among neighborhood characteristics, mobility limitation, and social participation in late life. J Gerontol B Psychol Sci Soc Sci. 2017. 10.1093/geronb/gbw215 PMC6377035 28158866

[46] Kuiper JS, Zuidersma M, Oude Voshaar RC, Zuidema SU, van den Heuvel ER, Stolk RP (2015). Social relationships and risk of dementia: a systematic review and meta-analysis of longitudinal cohort studies. Ageing Res Rev.

[47] Inagami S, Cohen DA, Finch BK. Non-residential neighborhood exposures suppress neighborhood effects on self-rated health Soc Sci Med 1982 2007;65(8):1779–1791. 10.1016/j.socscimed.2007.05.051 17614175

[48] Vallée J, Chauvin P (2012). Investigating the effects of medical density on health-seeking behaviours using a multiscale approach to residential and activity spaces: results from a prospective cohort study in the Paris metropolitan area, France. Int J Health Geogr.

[49] Baker PS, Bodner EV, Allman RM (2003). Measuring life-space mobility in community-dwelling older adults. J Am Geriatr Soc.

[50] Vallée J (2017). The daycourse of place. Soc Sci Med.

